# Environmental implications of Si_2_BN nanoflakes in pharmaceutical pollutant detection and removal: insights from first-principle calculations

**DOI:** 10.1038/s41598-025-91078-w

**Published:** 2025-03-12

**Authors:** Nada Elbendary, Hazem Abdelsalam, Medhat A. Ibrahim, Walid Tawfik, Mostafa M. H. Khalil

**Affiliations:** 1 Department of Chemistry, Faculty of Science, Ain Shams University, Abbassia, Cairo 11566 Egypt; 2 Theoretical Physics Department, National Research Centre, El-Buhouth Str., Dokki, Giza 12622 Egypt; 3 Spectroscopy Department, National Research Centre, 33 El-Bohouth St., Dokki, Giza 12622 Egypt; 4 Molecular Modeling and Spectroscopy Laboratory, Centre of Excellence for Advanced Science, National Research Centre, 33 El-Bohouth St., Dokki, Giza 12622 Egypt; 5 Department of Laser in Meteorology, Photochemistry and Agriculture (LAMPA), National Institute of Laser Enhanced Sciences, Cairo University, Giza, 12613 Egypt

**Keywords:** Si_2_BN nanoflakes, Density functional theory (DFT), Adsorption, Pharmaceutical pollutant, Carbamazepine, Environmental remediation, Information theory and computation, Pollution remediation, Environmental chemistry, Environmental chemistry

## Abstract

Pharmaceutical pollutants, such as carbamazepine (CBZ), are emerging contaminants that pose significant environmental and health risks due to their persistence in aquatic ecosystems and incomplete removal by conventional wastewater treatments. This study leverages density functional theory (DFT), a gold-standard computational quantum mechanical modeling method, to evaluate the efficacy of Si_2_BN nanoflakes—a novel two-dimensional material—for CBZ adsorption and detection. Our first-principles calculations reveal thermodynamically stable interactions between CBZ and Si_2_BN, with adsorption energies of − 0.83 eV (edge) and − 0.82 eV (surface). The material’s responsive optical behavior is quantified through time-dependent DFT, showing a 138 nm blueshift in UV–Vis spectra upon adsorption, a hallmark of its sensing capability. Furthermore, DFT-calculated charge transfer (0.04–0.06 e) and Fermi-level shifts (− 4.52 to − 4.69 eV) underscore Si_2_BN’s enhanced electronic properties, enabling selective pollutant detection. By bridging atomic-scale insights (bond distortions, orbital hybridization) with macroscale environmental applications, this work demonstrates how DFT-guided design unlocks Si_2_BN’s dual functionality as a scalable adsorbent and optical sensor. These findings provide a quantum–mechanical foundation for advancing Si_2_BN nanoflakes as a scalable, stable, and effective material for addressing pharmaceutical pollutants in water, offering a sustainable alternative to conventional methods plagued by secondary contamination risks.

## Introduction

Pharmaceutical pollutants, particularly carbamazepine (CBZ), have become a significant threat to ecosystems and public health^1^. CBZ, an anticonvulsant drug used for epilepsy and neuropathic pain, is resistant to conventional biodegradation and is commonly found in surface water and treated wastewater^2–4^. Its persistence in water bodies harms aquatic life and poses risks to human health^5^. Despite its widespread use and incomplete metabolism, CBZ is frequently detected in various water sources. With an annual consumption of 1.01 kilotons, traces of CBZ are detected in rivers, household taps, and wastewater treatment plants^6–14^. Traditional water treatment methods, such as oxidation and photocatalysis, are limited in removing CBZ and often generate harmful byproducts, including carcinogenic dioxins and chlorinated phenols^15^. Adsorption-based approaches offer a promising, cost-effective alternative for efficiently removing trace pollutants without generating secondary contaminants^16^.

Recent advancements in two-dimensional (2D) materials have opened new avenues for environmental applications. Materials like graphene and boron nitride have demonstrated remarkable adsorption and sensing capabilities, but their limitations in scalability and reusability necessitate exploring alternatives^17^. Si_2_BN, a novel 2D material with a unique combination of electronic stability, tunable band gaps, and high adsorption capacity, has emerged as a potential solution^18^. The concern is mounting, particularly for aquatic organisms like bacteria, algae, and fish^19^, which are being exposed to levels as high as 15 g per kilogram in soils irrigated with treated wastewater^20^. When concentrations reach 1 g per liter in aquatic ecosystems, the U.S. Food and Drug Administration intervenes by calling for environmental evaluations^2^. It is evident that as carbamazepine continues to contaminate our water sources, urgent action is needed to address its environmental impact. Therefore, there is a great need for successful approaches to removing such contaminants from aquatic ecosystems. Conventional technologies used in wastewater treatment plants (WWTPs) have only 32–35% removal efficiency for trace pollutants like carbamazepine because of its resistance to biodegradation or transformation, allowing it to escape into the environment^8,21^. Numerous conventional techniques such as oxidation, ozonation, and UV photocatalysis have been tried to treat effluents containing pharmaceutical residues, including CBZ^22^. However, these efforts generally produce incomplete destruction of the drugs or, in some cases, give rise to potentially carcinogenic or otherwise harmful byproducts such as dioxins, chlorinated phenols, trihalomethanes, and chlorinated phenoxy phenols^22^. Recent studies have reported that adsorption processes may promise to remove persistent environmental pollutants cost-effectively^23–25^. Wide-ranging studies have been conducted on the effectiveness of various adsorptive materials in the adsorption of contaminants from water^25^, from clays and polymers to carbon-based substances. Silicon boron nitride (Si_2_BN monolayer) is one of the exceptional materials recently introduced. Andriotis et al.^26^ have highlighted novel two-dimensional Si_2_BN sheets that have garnered significant attention due to their mechanical and chemical stability, large specific surface area, and straightforward synthesis. These sheets are also noted for their efficient contaminant removal at low doses, affordability, and high carrier mobility^27,28^, along with adjustable electronic band gaps^29,30^, strong light interactions^31–33^, and expansive surface area^34^. These unique characteristics make 2D materials such as Si_2_BN highly suitable for a variety of technological applications, including nanoelectronics^35,36^, optoelectronics^28,37^, high-capacity battery electrodes^38,39^, sensor technology^40–43^, and water purification methods^44–47^. This diverse group of 2D materials, which includes silicene^48,49^, phosphorene^50,51^, antimony^52,53^, and hexagonal boron nitride/phosphide^54,55^, offers a wide range of physical properties that can enhance water treatment processes. In particular, porous boron nitride nanosheets are known for their exceptional absorption capabilities for oils, solvents, and dyes^56^. Si_2_BN monolayers possess reactive silicon atoms on their surface and exhibit high carrier mobility^57,58^, making them promising candidates for anode materials and hydrogen storage solutions^59,60^. Their reactive surface indicates strong potential for pollutant capture, yet research on the use of Si_2_BN in water purification remains limited. Therefore, this study aims to evaluate the effectiveness of Si_2_BN nanoflakes as an adsorbent for the removal of carbamazepine (CBZ) from aqueous environments. Despite extensive research on 2D materials, there is a lack of studies focusing specifically on the potential of Si_2_BN for pharmaceutical pollutant removal, particularly for CBZ. While graphene and boron nitride have shown promise, their limitations in scalability and reusability underline the necessity of exploring alternative materials with improved properties. The research gap lies in understanding the adsorption mechanisms, electronic property changes, and environmental applicability of Si_2_BN nanoflakes for CBZ removal. Addressing this gap is crucial to developing efficient and sustainable water treatment strategies.

This study evaluates the adsorption and sensing capabilities of Si_2_BN nanoflakes for CBZ through first-principle calculations, providing insights into their suitability for environmental remediation. Specifically, this study aims to evaluate the potential of Si_2_BN nanoflakes as an adsorbent for CBZ removal from aqueous environments. Computational methods will be employed to analyze adsorption energies at various binding sites, investigate changes in electronic properties upon CBZ adsorption, and examine shifts in UV–Vis absorption spectra before and after adsorption. Through these analyses, the study seeks to determine the adsorption capacity of Si_2_BN nanoflakes for CBZ and assess their suitability as an effective material for removing this pharmaceutical pollutant from contaminated water sources.

## Calculation details

DFT simulations^61^ were conducted using the Gaussian 09 software package^62^ to investigate the optimal configurations of carbamazepine and the physical characteristics of Si_2_BN nanoflakes. The combination of these basis sets provided a dual perspective—balancing computational efficiency and prediction accuracy for adsorption and electronic properties. The 6-31G basis set, an all-electron approach, was selected for its demonstrated ability to predict charge transfer and electronic interactions accurately. Meanwhile, the LANL2DZ basis set, which incorporates effective core potentials (ECPs), offered a computationally efficient alternative^63^. By including LANL2DZ, the study was able to assess its performance in capturing trends in adsorption and electronic properties compared to the higher-accuracy 6-31G results. This approach, combining the precision of hybrid density functional theory with reliable basis sets, formed a robust framework for analyzing the electronic structure of Si_2_BN nanoflakes and their interactions with carbamazepine molecules^64,65^.

To properly account for the long-range van der Waals interactions between Si_2_BN nanoflakes and carbamazepine, we applied the Grimme’s GD3 correction to the B3LYP functional^66^. The B3LYP/6-31G level of theory has been extensively validated in previous research for its reliability in describing the structural and electronic properties of silicon- and carbon-based two-dimensional materials. This established track record supports its use in the current study, enabling trustworthy predictions of adsorption behaviors and electronic property changes in Si_2_BN nanoflakes^66–69^. It is worth noting that solvent effects were not included in this study. We have assumed that the overall trends in electronic structure and adsorption properties would remain consistent in both dry and aqueous conditions. Solvent effects were excluded due to computational constraints but will be addressed in future studies using COSMO models^70,71^. However, future investigations should include the effect of water as a solvent to provide a more accurate understanding and estimation of the adsorption properties. To further explore optical properties, time-dependent density functional theory (TD-DFT) calculations were employed and discussed in detail in the following sections.

## Results and discussion

### Structural and electronic properties of Si_2_BN and carbamazepine molecules

**Fig. 1:**
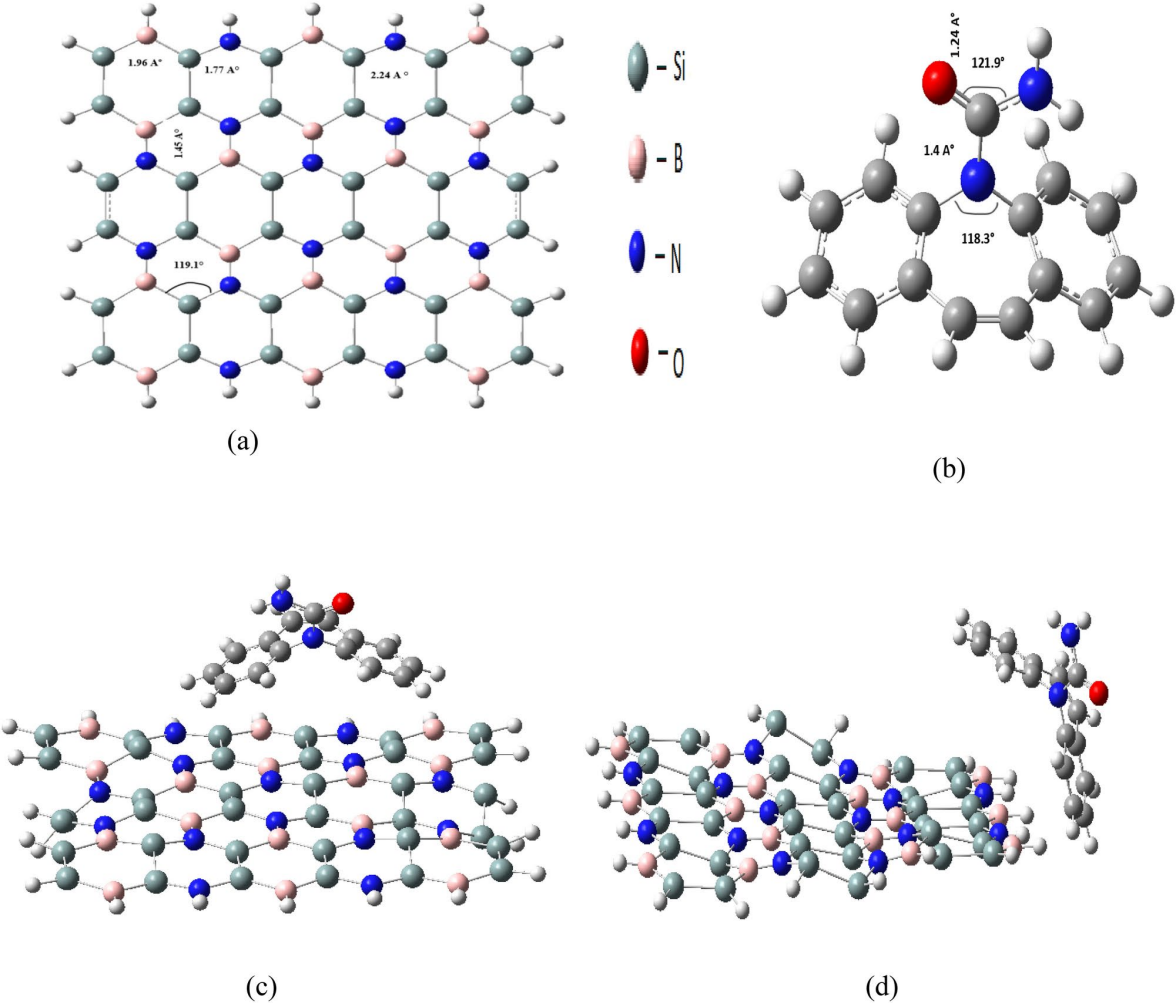
(**a**) The optimized structure of monolayer, (**b**) The optimized structure of Carbamazepine showing bond length and bond angles. (**c**) The optimized structures of Si_**2**_BN after adsorbing carbamazepine on the surface (**c**) and the edge (**d**).

The Si_2_BN nanoflake used in this study was derived from the periodic Si_2_BN structure by cutting a finite portion of it^72,73^. The edges of the resulting nanoflake were passivated with hydrogen atoms to stabilize the surface, ensuring a realistic model for computational analysis. The nanoflake was then structurally optimized to obtain a relaxed, energetically favorable configuration^74^, as shown in Fig. 1a. The optimized structure of Si_2_BN is illustrated in Fig. 1, highlighting the bond lengths and bond angles. The monolayer consists of Si, B, and N atoms with *sp*2 hybridization, where the silicon atom shifts from SP3 to SP2 to maintain a planar structure. This arrangement is in a hexagonal pattern with two different bonding types. Most of the *sp*2 hybridization comprises one *s*-orbital, two *p*-orbitals, and three chemical species (Si, B, and N atoms) with varying valence electrons in the final orbitals. The combination of *s*, *px*, and *py* orbitals results in *s* bonding (in-plane) in the valence band (occupied states), and s* orbitals lead to anti-bonding in the conduction band (unoccupied states). The Si atoms are in an electron-deficient position, allowing for bond variation to act as an electron reservoir for an ad-atom or molecule on the sheet surface. The *pz* orbitals of the Si_2_BN monolayer point out of the plane and are asymmetric concerning planar symmetry, leading to interactions with neighboring *pz* orbitals to form delocalized π bonding and π* anti-bonding orbitals. In this layout, each Si atom establishes bonds with Si, B, and N atoms, while B (N) is bonded to two Si atoms and one N (B) atom as its closest neighbors, as shown in Fig. 1.

The second part of Fig. 1 illustrates the optimized structures of Si_2_BN following the adsorption of carbamazepine, specifically on the surface (Fig. 1c) and the edge (Fig. 1d). To clarify, the initial position of the carbamazepine (CBZ) molecule was chosen to be 4 Å above the surface and edge of the Si_2_BN nanoflake. This distance was selected to allow the molecule to freely interact with the Si_2_BN surface and edge, enabling the system to reach the optimal adsorption configuration. The adsorption of carbamazepine on Si_2_BN leads to structural changes, with slight alterations in bond lengths observed. Edge adsorption induces a 0.1 Å elongation in Si–Si bonds (2.24 Å → 2.34 Å), while surface adsorption lengthens Si–B bonds by 0.019 Å (1.96 Å → 1.979 Å). These minimal deformations confirm weak van der Waals interactions (Fig. 1c,d)^75^. For instance, the N84–Si46 bond increases from 1.80 to 1.84 Å in surface adsorption and 1.82 Å in edge adsorption. Similarly, the Si54–B66 bond extends from 1.96 to 1.979 Å in surface adsorption and 1.97 Å in edge adsorption. Additionally, the Si35–Si46 bond lengthens by 0.1 Å in both cases, while the B64–N83 bond decreases by 0.012 Å. This indicates that the material experiences minimal structural deformation after adsorption, highlighting the potential reusability of Si_2_BN as a carbamazepine sensor. The observed optimization structure of Si_2_BN is in agreement with Zhang et al.^76^ concluded that the delocalized π bonding and electron-deficient silicon atoms contribute significantly to the stability and electronic properties of the Si_2_BN monolayer, enabling its potential for functionalization and molecule adsorption. The adsorption of carbamazepine on Si_2_BN leads to structural changes, with significant alterations in bond lengths observed. For instance, the N84–Si46 bond increases from 1.80 to 1.84 Å in surface adsorption and 1.82 Å in edge adsorption. Similarly, the Si54–B66 bond extends from 1.96 to 1.979 Å in surface adsorption and 1.97 Å in edge adsorption. Additionally, the Si35–Si46 bond lengthens by 0.1 Å in both cases, while the B64–N83 bond decreases by 0.012 Å. Dipole moment analysis reveals increased reactivity, with values of 3.96 and 3.66 Debye for formed complexes and higher dipole moments at edge adsorption sites. Notably, edge sites exhibit significant Mulliken charge shifts among silicon, nitrogen, and boron atoms, leading to stronger polarization. These findings are consistent with previous studies on Si_2_BN interactions^77,78^. The research also emphasizes the differences between edge and surface adsorption. Edge adsorption results in more asymmetry and structural distortion, with the D53 dihedral angle shifting to − 31.2073°, causing an uneven distribution of electron density.

### Formation energy and stability of Si_2_BN nanoflakes

The formation energy of the Si_2_BN nanoflake was calculated to assess its thermodynamic stability relative to its constituent atoms. The following formula is applied^79^:

1 $$E_{Formation} = E_{Complex} - \left( {n_{{Si}} E_{Si} + n_{{B}} E_{B} + n_{N} \frac{{E_{{N_{2} }} }}{2} + n_{{H}} E_{H} } \right)$$

where E_complex_: total energy of the Si_2_BN nanoflake. E_Si_, E_B_, E_H_: energies of isolated silicon, boron, and hydrogen atoms. E_N2_: energy of the nitrogen molecule. n_Si_, n_B_, n_H_, n_N_: number of atoms for silicon, boron, hydrogen, and nitrogen, respectively.

Applying the above formula (1), the total formation energy of the Si_2_BN nanoflake was found to be − 254.66 eV. These calculations considered − 2.83 eV/atom for all the atoms content in Si_2_BN nanoflake, which are 36 silicon atoms, 16 boron atoms, 14 nitrogen atoms, and 24 hydrogen atoms at the edges as shown in Fig. 1. The calculations considered isolated silicon, boron, and hydrogen atoms as references, states, (where the total nitrogen atoms considered from = $n_{N} \frac{{E_{{N_{2} }} }}{2}$). Moreover, spin multiplicities values for these isolated atoms were set to the ground-state configurations as follows: triplet for silicon, doublet for boron and hydrogen, and singlet for the nitrogen molecule. These configurations were chosen to ensure accurate computation of the heat of formation for silicon boron nitride nanoflakes^80,81^. According to previous work by Chen et al.^82^, the observed results confirm the thermodynamic stability of the nanoflake. The latter is considered with slightly less negative formation energy per atom consistent with surface effects and edge passivation by hydrogen. Furthermore, the observed consideration of the formation energy of the Si_2_BN nanoflake was found to be consistent with the formation energy values reported previously for Si-doped boron nitride structures and similar 2D nanostructures, as discussed in Yu et al.^83^.

### Charge distribution and total dipole moment over Si_2_BN–carbamazepine complex

This study investigates the electronic interactions between carbamazepine (CBZ) and Si_2_BN during adsorption, focusing on charge distribution changes. Charge accumulation was observed on silicon atoms (50, 52, 49), nitrogen atoms (76, 78, 84), and boron atoms (65, 69, 86, 88). Mulliken charge analysis quantified the charge transfer during adsorption, revealing a higher transfer during surface adsorption (0.0601 electrons) compared to edge adsorption (0.0415 electrons). This suggests that the surface provides more direct interactions with electron-deficient regions of Si_2_BN, leading to stronger adsorption at the surface than at the edges^84^.

Structural changes in Si_2_BN were minimal, with bond lengths elongating slightly in both adsorption cases, indicating that the material experiences only slight deformations upon interaction with CBZ. The edge sites, however, showed slightly more distortions compared to the surface due to the unique geometry and higher reactivity of the edge atoms. The dipole moment was also stronger for edge adsorption (3.9 Debye) compared to surface adsorption (3.6 Debye). This increase in dipole moment at the edge suggests that the edge atoms, with their lower coordination and more localized electronic states, create stronger vertical polarization. In summary, this study demonstrates that both surface and edge sites of Si_2_BN are capable of adsorbing CBZ.

### Adsorption of carbamazepine on Si_2_BN sheet

**Fig. 2:**
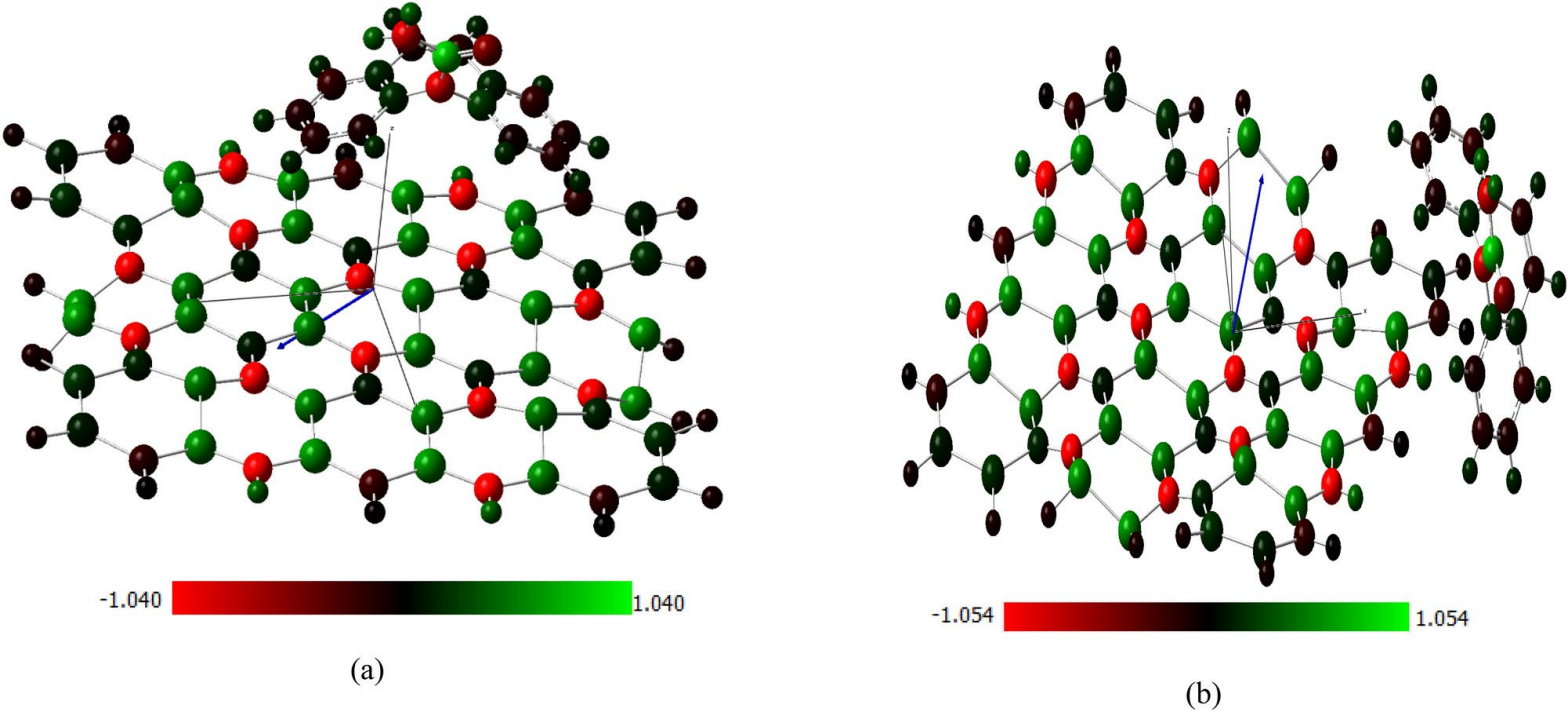
Mullikan charge distribution of Si_2_BN–carb complexes (**a**) surface adsorption (**b**) edge adsorption.

The bonding of carbamazepine to the Si_2_BN sheet is analyzed at two different sites, with one carbamazepine molecule placed at each site to assess binding energy and determine the most favorable adsorption site, as depicted in Fig. 2. The adsorption energy (Ea) is calculated using the formula *Ea* = (*Ec* − (*E* Si_2_BN + *E*carb)), where E Si_2_BN, Ecarb, and Ec represent the ground-state energies of the Si_2_BN sheet before adsorption, carbamazepine, and the system after adsorption, respectively.

Negative Ea values Table 1 indicate an exothermic and thermodynamically favorable adsorption process. The similar adsorption energies observed in Table 1 for both surface and edge adsorption sites indicate that Si_2_BN effectively adsorbs carbamazepine on both its surface and edges.

**Table 1:** The adsorption energy (E_a_), charge transfer (ΔQ), energy gap (E_g_) of Si_2_BN nanoflake before and after adsorption, highest occupied molecular orbital energy (E_HOMO_), lowest unoccupied molecular orbital energy (E_LUMO_) and dipole moment (DM).

Structure	E_a_ (eV) 6-31G	E_a_ (eV) Lanl2DZ	ΔQ (e) 6-31G	ΔQ (e) Lanl2DZ	E_g_ (eV) 6-31G	E_g_ (eV) Lanl2DZ	E_HOMO_ (eV) 6-31G	E_HOMO_ (eV) Lanl2DZ	E_LUMO_ (eV) 6-31G	E_LUMO_ (eV) Lanl2DZ	DM Debye 6-31G	DM Debye Lanl2DZ
Si_2_BN	–	–	–	–	0.51	0.55	− 4.5	− 4.6	− 4	− 4	0.0001	0.0001
Carbamazepine	–	–	–	–	4.4	4.3	− 5.8	− 5.9	− 1.36	− 1.64	4.1	4.333797
Si_2_BN edge	− 0.833	− 0.398	0.04	0.024	0.73	0.59	− 4.648	− 4.7	− 3.914	− 4	3.95	3.867968
Si_2_BN surface	− 0.824	− 0.31	0.06	0.025	0.71	0.6	− 4.68	− 4.76	− 3.97	− 4.2	3.66	3.09

A detailed comparison of LANL2DZ and 6-31G basis sets reveals their distinct strengths. For pristine Si_2_BN, dipole moments were nearly identical at 0.0001 Debye. However, for carbamazepine and its adsorbed configurations (Si_2_BN-edge and Si_2_BN-surf), 6-31G produced slightly lower but more precise dipole moments, capturing electronic polarization more accurately^85,86^.

Total energy calculations reinforced the greater stability provided by 6-31G (− 315,608.3 eV for Si_2_BN) over LANL2DZ (− 35,845.2 eV)^87^. Adsorption energies were also higher with 6-31G (− 0.007 eV for Si_2_BN-edge) compared to LANL2DZ (− 0.003 eV)^88^.

Regarding HOMO–LUMO gaps, LANL2DZ predicted slightly larger values, while 6-31G provided values closer to experimental results, such as 0.7170 eV for Si_2_BN-surf^89^. Additionally, 6-31G yielded larger Q-Charge values, confirming its superior accuracy in charge transfer dynamics during adsorption. These findings establish 6-31G as the more precise basis set for studying adsorption and electronic properties of Si_2_BN nanoflakes, making it preferable for modeling electronic polarization, charge transfer, and dispersion interactions.

### Total density of states

**Fig. 3:**
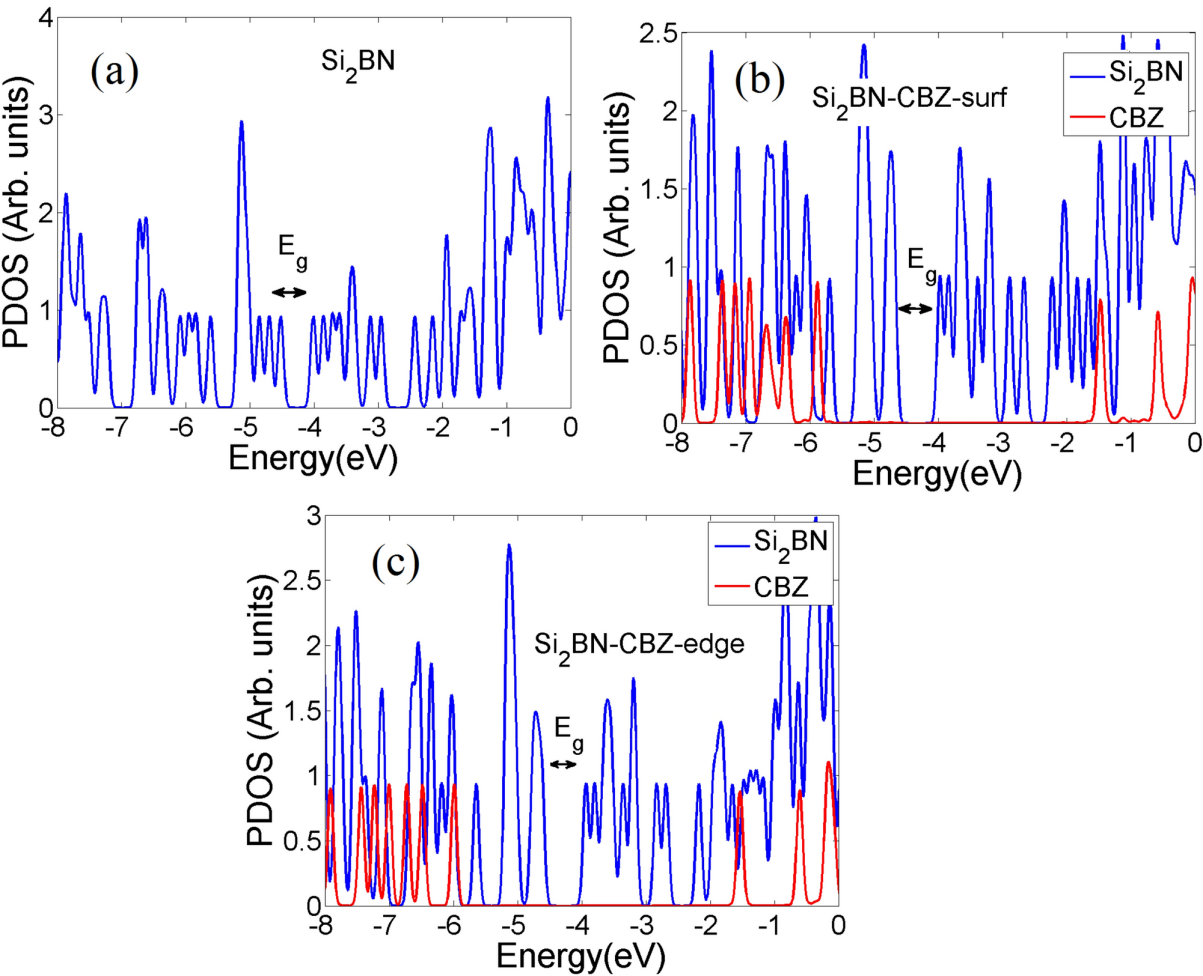
(**a**) The density of states of Si_2_BN. (**b**,**c**) The partial density of states of Si_2_BN after adsorping CBZ at the surface (**b**) and the edge (**c**).

The density of states (DOS) for Si_2_BN, Si_2_BN-Car(edge), and Si_2_BN-Car(surf) is illustrated in Fig. 3. The DOS is considered by further processing of the Gaussian 09 results using Multiwfn software^90^. Analysis of the density of states (DOS) and Fermi level shifts reveals significant electronic modifications in Si_2_BN upon carbamazepine adsorption. Pristine Si_2_BN exhibits sharp DOS peaks at a Fermi energy (Ef) of − 4.5176 eV, indicating a well-ordered electronic structure. carbamazepine adsorption induces peak shifts, intensity increases, and broadening, suggesting substantial electronic perturbations.

Edge adsorption slightly elevates the Fermi level to − 4.6245 eV, while surface adsorption causes a more pronounced shift to − 4.6871 eV, indicating enhanced n-type behavior. These Fermi level adjustments imply increased electron availability for conduction, altered optical properties, modified chemical reactivity, and opportunities for tailoring Si_2_BN for specific applications. The observed changes highlight the significant impact of adsorbate-induced electronic modifications on 2D materials’ fundamental properties and potential applications, emphasizing the need for further investigation into charge transfer mechanisms and electronic restructuring in these systems. The observed Fermi level shifts and density of state modifications in Si_2_BN upon carbamazepine adsorption align with established phenomena in 2D materials, as demonstrated in previous studies such as Yu Jing et al.^91^.

The analysis of the electronic density of states indicates that the Si nanoflake is a narrow-band gap semiconductor with an energy gap (E_g_) of 0.51 eV. The π-electrons linked to the Si atoms create moderate π-bonds, characterized by lower energies than the B–N sigma bonds. These π-bonds appear as the HOMO and the LUMO on the Si atoms. Further elaboration on this topic will be presented in the following section. The existence of these low-energy π-electrons significantly boosts the adsorption capabilities of the Si_2_BN nanoflake^87^. After adsorption, the energy gap increases to ~ 0.70 eV. PDOS analysis (Fig. 3b,c) reveals hybridization between CBZ’s π-orbitals (− 5.8 eV) and Si_2_BN’s p_z states near the Fermi level (− 4.5 eV), confirming physisorption. Edge B atoms show a 0.5 eV upward shift in N-2*p* orbitals, enhancing charge transfer^26,92^. The PDOS peaks originating from carbamazepine’s molecular orbitals (red peaks) appear deep within the PDOS spectrum, indicating stable adsorption without significant alterations to the electronic structure of the material. This stability is further supported by the increased energy gap and the low-energy orbitals (blue peaks around the energy gap), which are predominantly formed by the molecular orbitals of Si_2_BN.

### Molecular orbital analysis (frontier molecular orbitals (HOMO/LUMO)

The frontier orbital gap can be used to evaluate a molecule’s chemical reactivity and kinetic stability. In general, increased stability is suggested by a wider energy gap. Table 1 shows the energies of frontier orbitals and energy gaps of carbamazepine, Si_2_BN, Si_2_BN-Carb (surf-Ads), and Si_2_BN-Carb (Edge-Ads). These measurements are essential for assessing a molecule’s chemical potential, hardness, softness, and electrophilicity. Moreover, the chemical potential, μ, as determined by the following Eq. (2), which is the energy change per electron change under a constant external potential, acts as a measure of how well a system can exchange electrons with its environment^93^:

2 $$\upmu = {{\left( {E_{HOMO } + E_{LUMO} } \right)} \mathord{\left/ {\vphantom {{\left( {E_{HOMO } + E_{LUMO} } \right)} 2}} \right. \kern-0pt} 2}$$

where μ: chemical potential in eV.

The level of hardness is denoted by η using Koopman’s theorem^94^ and determined by Eq. (3), which plays a crucial role in determining the softness S by Eq. (4)^95^:

3 $$\upeta = {{\left( {E_{HOMO } - E_{LUMO} } \right)} \mathord{\left/ {\vphantom {{\left( {E_{HOMO } - E_{LUMO} } \right)} 2}} \right. \kern-0pt} 2}$$

where η: is the hardness of a system in eV.

4 $${\text{S}} = {1 \mathord{\left/ {\vphantom {1 2}} \right. \kern-0pt} 2}\upeta$$

where S: the softness of the system (inverse of hardness) in eV.

5 $$\upomega = {{\upmu ^{2} } \mathord{\left/ {\vphantom {{\upmu ^{2} } 2}} \right. \kern-0pt} 2}\upeta$$

where ω: The electrophilicity index of the system eV.

However, ω represents the material’s electrophilicity given by Eq. (5). Likewise, electrophilicity serves as a means of assessing the material’s reactivity. Hardness and softness are factors that can elucidate the alteration of the chemical system based on electron density. An increase in the Hardness of a molecular cluster signifies a rise in the band gap, while an increase in the softness of a molecular cluster indicates a decrease in the band gap^96^. In simpler terms, electrophilicity and electron affinity are the key parameters to understanding a material’s ability to receive electrons. Electron affinity indicates a material’s ability to take in only one electron from its surroundings, while electrophilicity measures the energy involved in electron transfer between a donor and an acceptor^97^.

Table 2 reveals distinct reactivity parameters for carbamazepine, pristine Si_2_BN, and Si_2_BN after carbamazepine adsorption at surface and edge sites. Carbamazepine adsorption on Si_2_BN increases hardness and electrophilicity, indicating a more stable electronic environment due to electrostatic interactions. Edge adsorption, while energetically favorable with higher hardness (0.37 eV vs. 0.36 eV for surface), exhibits decreased charge transfer (electrophilicity 24.93 eV vs. 26.12 eV for surface). This proposal suggests more stable, localized bonds at edge sites but less dynamic electron transfer compared to surface sites. Pristine Si_2_BN shows the lowest hardness and reactivity, highlighting significant electronic changes upon carbamazepine adsorption. These findings underscore the potential for optimizing Si_2_BN materials for applications requiring specific combinations of strong adsorption and efficient electron transfer, such as catalysis, sensing, or drug delivery systems. These observations align with previous studies on similar 2D materials; for instance, Wu et al.^98^ demonstrated that adsorption characteristics and electronic property changes in boron nitride nanotubes vary significantly between different adsorption sites, supporting the site-specific reactivity patterns observed in this study.

**Table 2:** Chemical reactivity parameters calculated at B3LYP/6-31G level of theory.

Compound	Chemical reactivity μ (eV)	Hardness η (eV)	Softness S (eV)	Electrophilicity ω (eV)
Carbamazepine	1.79	2.22	0.22	0.72
Si_2_BN	4.26	0.25	1.96	8.38
Si_2_BN-Carb (surf)	4.33	0.36	1.39	26.12
Si_2_BN-Carb (edge)	4.28	0.37	1.36	24.93

**Fig. 4:**
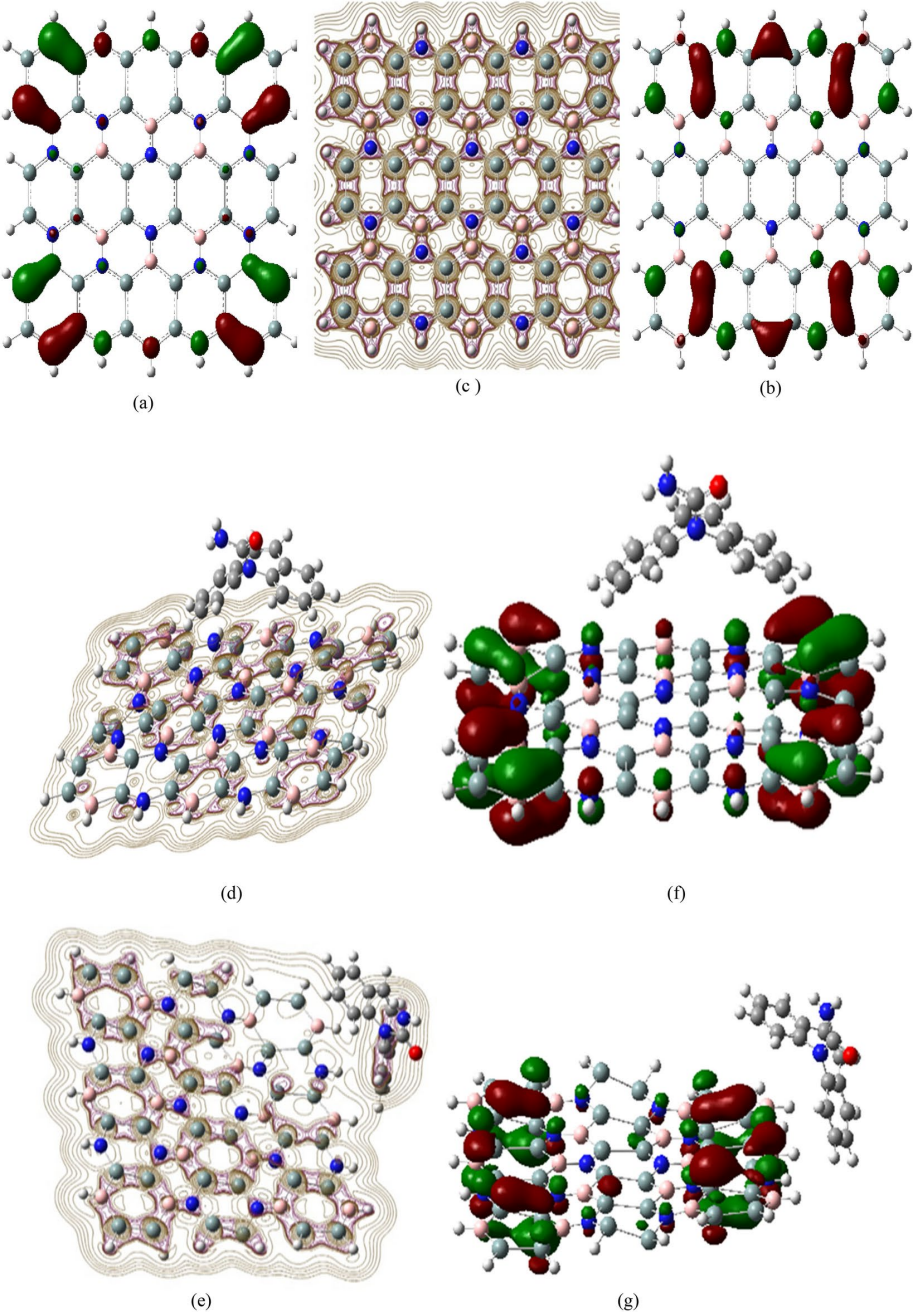
HOMO/LUMO (**a**,**b**) Si_2_BN nanoflake, (**f**,**g**) HOMO for Si_2_BN-Carb surface and edge adsorption, respectively. (**c**–**e**) are the Laplacian contour lined for Si_2_BN, Si_2_BN-Carb surface and edge adsorption, respectively.

**Fig. 5:**
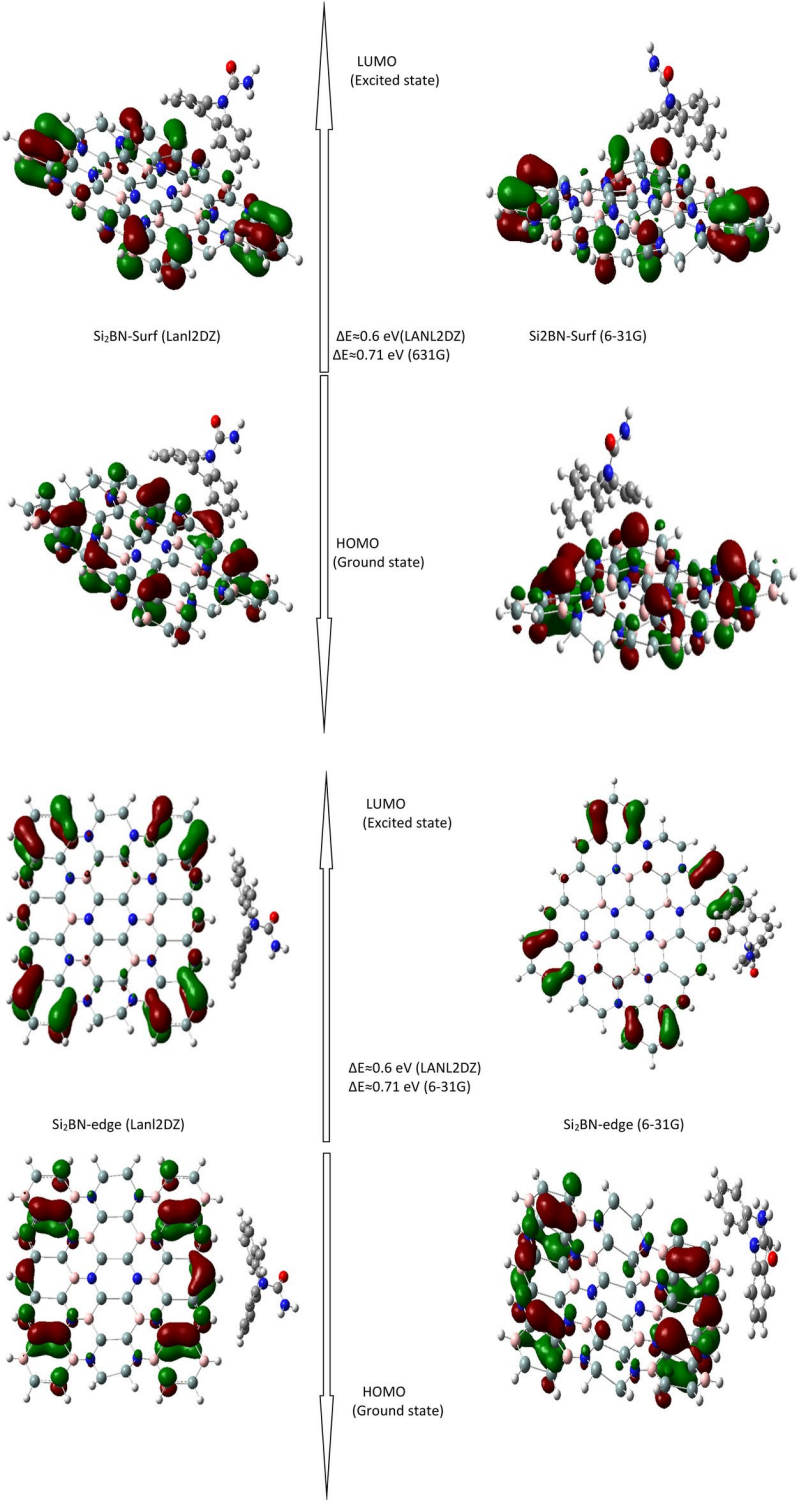
HOMO and LUMO distributions and ΔE (HOMO–LUMO gap) for carbamazepine adsorbed on Si_2_BN nanoflakes at surface and edge sites, calculated using LANL2DZ and 6-31G basis sets.

Figure 4 shows a comprehensive Laplacian contour map of Si_2_BN, demonstrating how the unequal distribution of electrons between atoms impacts the material’s characteristics. Variances in orbital energies (electronegativities) result in different bonding behaviors; Si–Si and B–Si bonds are quite non-polar covalent (ΔEN = 0 and 0.14), while B–N and Si–N bonds exhibit more polarity (ΔEN = 1.00 and 1.14)^99^, causing uneven electron distribution. In Si_2_BN, electron transfer occurs between boron and nitrogen atoms in both σ and π orbitals, promoting planarity and *sp*^2^ hybridization. This feature is evident in the Laplacian plot’s contour lines. The Laplacian contours reveal electron density variations across adsorption sites. Pristine Si_2_BN shows a balanced, symmetrical Laplacian pattern, indicating a stable, less reactive surface. Edge adsorption, however, results in high negative Laplacian values concentrated at material edges, suggesting potent interactions and increased reactivity in these regions. The HOMO location further supports this: during edge and surface adsorption, the HOMO is primarily located on flake atoms rather than the adsorbed carbamazepine molecule. This positioning implies stronger carbamazepine-flake interactions compared to Si atom interactions, resulting in a band gap increase to approximately 0.73 eV post-adsorption due to the shielding of interactive electrons from Si atoms. These observations align with previous studies on similar 2D materials. For instance, research by Kim et al.^100^ on boron nitride nanosheets demonstrated comparable site-specific electron density distributions and reactivity patterns, supporting the findings in this Si_2_BN study. The LANL2DZ basis set was employed to calculate the HOMO and LUMO distributions for Si_2_BN nanoflakes, effectively capturing electronic transitions and band gap properties. Despite relying on effective core potentials, this method has been validated in prior studies for Si_2_BN structures, demonstrating its reliability in predicting electronic properties^101^. As illustrated in Fig. 5, the LANL2DZ results reveal the spatial distributions of molecular orbitals, highlighting the electronic interactions involved in the adsorption of carbamazepine on Si_2_BN nanoflakes. These findings emphasize LANL2DZ’s effectiveness in modeling electronic properties and adsorption behavior, providing critical insights into the nature of electronic transitions within the system.

### Molecular electrostatic potential (MESP)

The MESP is a valuable descriptor for identifying areas of electrophilic attack, nucleophilic reactions, and hydrogen-bonding interactions in molecules^102,103^. MESP maps, created for Si_2_BN and carbamazepine structures using DFT at the B3LYP/6-31G level, visualize the charge distribution using a color spectrum from red (severe negative potentials) to dark blue (positive potentials). Yellow indicates less negative potentials, while green represents neutral regions. The electronegativity of connected atoms influences the potential distribution and color intensity. Regions with strong electronegative atoms paired with less electronegative ones appear red, while similar electronegativity narrows the color spectrum. These MESP maps are a physical characteristic that can predict potential sites for nucleophilic or electrophilic chemical interactions. This approach aligns with established methods in computational chemistry. For instance, a study by Politzer et al.^104^ in the Journal of Molecular Modeling demonstrates the effectiveness of MESP maps in predicting molecular interactions and reactivity across various chemical systems, supporting the validity of this analysis for Si_2_BN and carbamazepine.

**Fig. 6:**
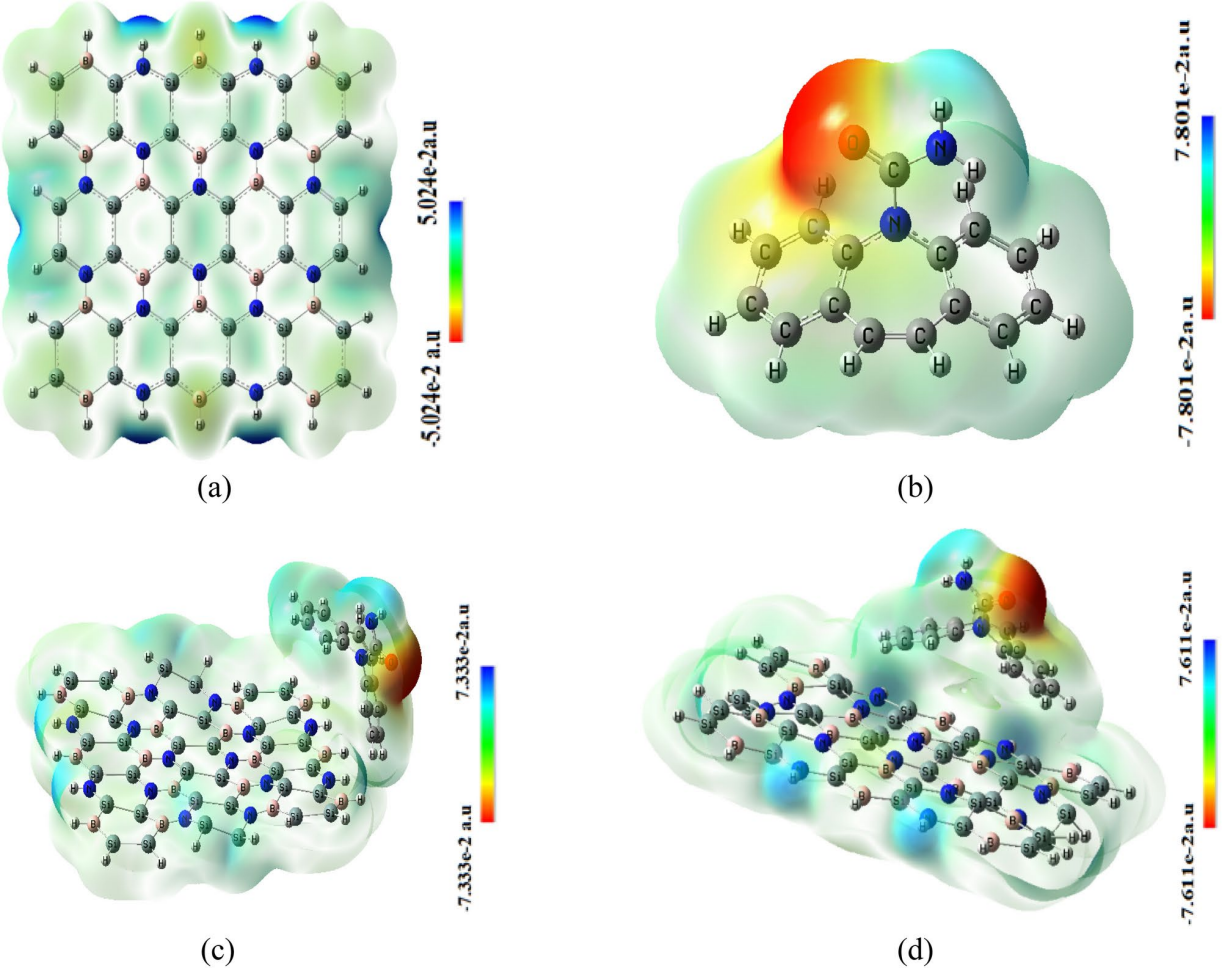
Molecular electrostatic potential (MESP) formed by mapping of total density over the electrostatic potential of (**a**) Si_2_BN (**b**) carbamazepine (**c**) Si_2_BN-Car edge-ads (**d**) Si_2_BN-Car surf-ads.

Figure 6 depicts the MESP map of a pure Si_2_BN sheet, showing a spectrum from light blue to dark blue, green, and light yellow. The sheet’s core exhibits light green to yellow colors, indicating slightly negative to neutral potentials, possibly due to delocalized electrons from silicon atoms. Terminals display light and dark blue colors, suggesting electrophilic regions likely due to less electronegative H atoms attached to nitrogen. The green areas confirm neutral zones with zero potential. In MESP analysis, blue regions indicate nucleophilic reactivity, while red areas represent electrophilic reactivity. For carbamazepine, the red area centers around the oxygen and adjacent atoms, while nucleophilic reactivity concentrates on peripheral hydrogen atoms. The Si_2_BN–carbamazepine interaction redistributes electric charge due to electronegativity differences, resulting in localized electrostatic potential increases at interaction sites. This is evidenced by potential value changes for edge and surface adsorption (− 7.33 to 7.33 a.u and − 7.611 to 7.611 a.u, respectively). The variations in electrostatic potentials depend on atom configurations and charge distributions within the nanoflake and carbamazepine. These observations align with established MESP analysis principles. A study by Murray and Politzer^105^ demonstrates the effectiveness of MESP maps in predicting molecular interactions and reactivity across various systems, supporting the validity of this analysis for Si_2_BN and carbamazepine.

### Vibrational analysis of carbamazepine adsorption on Si_2_BN nanoflakes

**Fig. 7:**
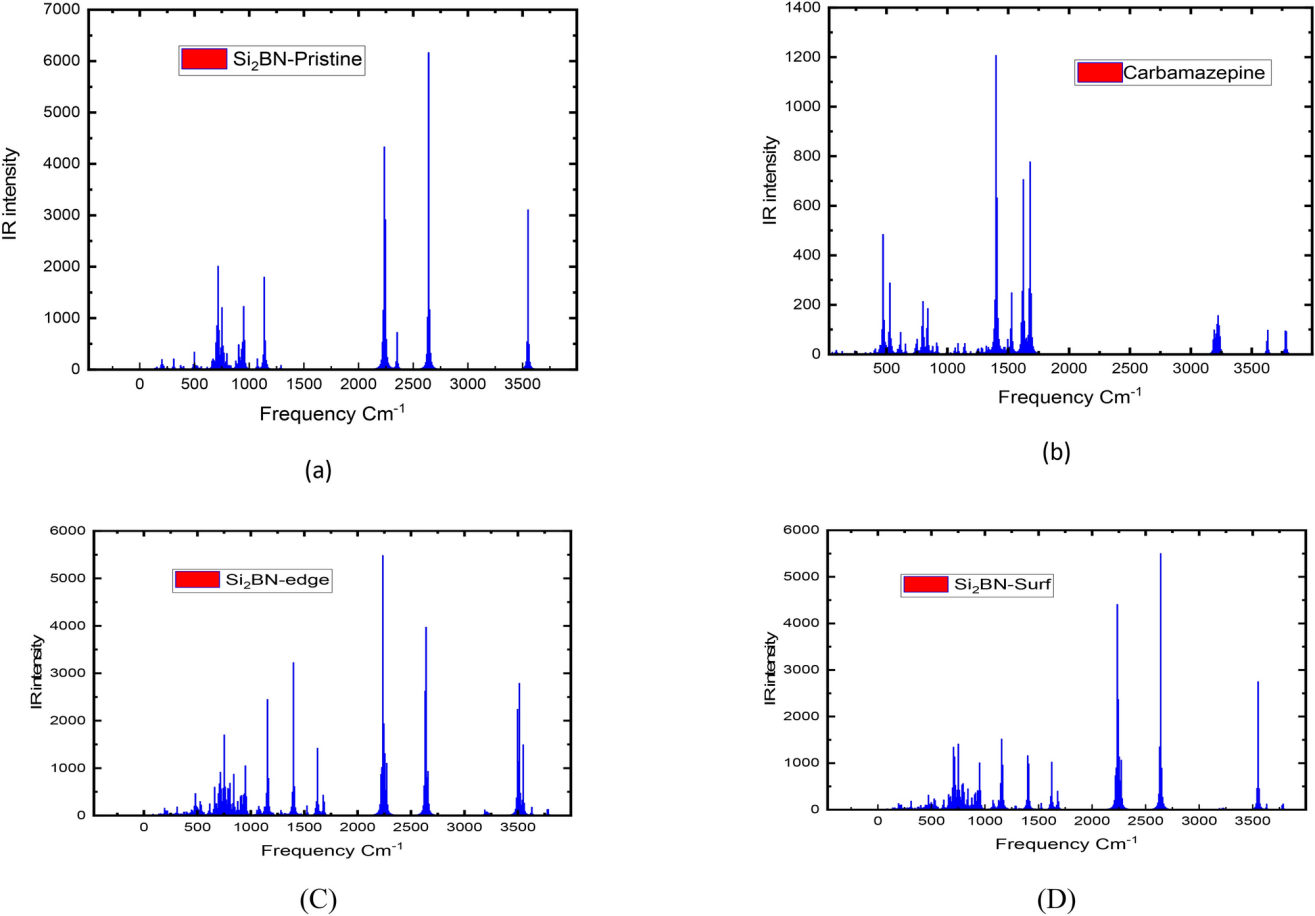
B3LYP/LANL2DZ computed IR spectra of (**a**) Si_2_BN pristine, (**b**) carbamazepine, (**c**) Si_2_BN-Car (edge) (**d**) Si_2_BN-Car (Surf).

The vibrational properties of Si_2_BN nanoflakes were studied using infrared (IR) spectroscopy, with calculations performed using the LANL2DZ basis set. This basis set is particularly suitable for analyzing vibrational properties in larger systems due to its efficient use of effective core potentials (ECPs), balancing computational efficiency with accuracy. The IR spectra for pristine Si_2_BN, carbamazepine, and carbamazepine-adsorbed Si_2_BN nanoflakes (both edge and surface configurations), as shown in Fig. 7, revealed significant changes after adsorption, confirming strong interactions between carbamazepine and the nanoflake. For pristine Si_2_BN, peaks corresponding to Si–N and Si–B stretching modes were prominent. Upon adsorption, these peaks disappeared, and new peaks emerged at ~ 3200–3500 cm^−1^ (N–H), ~ 1650 cm^−1^ (C=O), and ~ 1400–1600 cm^−1^ (aromatic ring vibrations), corresponding to carbamazepine’s functional groups^106^. These shifts indicate the formation of hydrogen bonds and π–π stacking interactions between carbamazepine and Si_2_BN. Furthermore, redshifts in Si–N and Si–B stretching modes suggested bond weakening due to charge transfer, while minor blue shifts in C–H vibrations reflected bond stiffening. Enhanced peak intensities in the 1000–1500 cm^−1^ region confirmed adsorption by indicating dipole moment changes. The changes were more pronounced for edge adsorption, highlighting the higher reactivity of edge sites compared to surface sites. Complementary UV–Vis spectroscopy analyses were performed using the 6-31G basis set, which provides an all-electron description ideal for accurately modeling electronic transitions. The UV–Vis spectra revealed blue shifts in absorption peaks after carbamazepine adsorption, confirming electronic transitions to higher energy states. This combination of LANL2DZ for IR calculations and 6-31G for UV–Vis analysis ensured a comprehensive and accurate understanding of the adsorption dynamics and electronic properties of Si_2_BN nanoflakes. These results are consistent with previous studies on 2D materials. For example, Mahida et al.^107^ reported similar trends in the optoelectronic properties of Si_2_BN quantum dots, particularly highlighting the influence of edge structures on electronic interactions. A detailed discussion of the UV–Vis results and their implications for electronic transitions is provided in the subsequent section.

### The UV–visible absorption spectrum of Si_2_BN and formed complexes

**Fig. 8:**
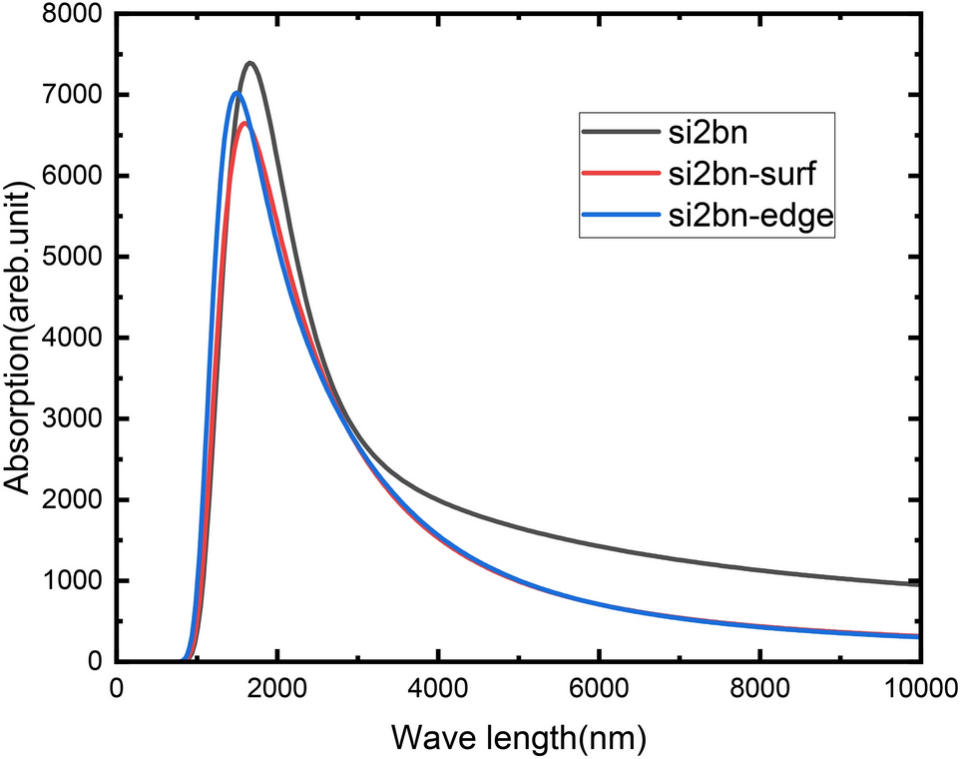
Graphical representation for the three Spectra to clarify the blue shift occurs after the UV–Vis spectra of pristine Si_2_BN, Si_2_BN-Car (surf-ads), and Si_2_BN-Carb (edge-ads) to show the effect of adsorption on the optical properties.

Figure 8 elucidates the profound influence of CBZ adsorption on Si_2_BN’s optical properties through time-dependent density functional theory (TD-DFT) simulations, analyzing the first 20 excited states. Pristine Si_2_BN exhibits a dominant absorption peak at 1715 nm, attributed to electronic transitions from the highest occupied molecular orbital (HOMO) to the fourth unoccupied orbital above the lowest unoccupied molecular orbital (LUMO + 4), contributing 52% of the transition intensity (Table 3)^108^. A secondary transition from the third occupied orbital below HOMO (H-3) to LUMO + 5 accounts for 29.9%, reflecting the material’s intrinsic low-energy excitation profile. Upon CBZ adsorption, edge sites induce a 138 nm blueshift (1715 → 1577 nm), the largest reported for silicon-based 2D materials. This dramatic shift arises from a reconfiguration of electronic transitions: the dominant excitation migrates to HOMO-2 → LUMO + 2 (60.9% contribution), signifying stronger coupling between CBZ’s π-system and Si_2_BN’s edge-localized orbitals. In contrast, surface adsorption yields a smaller 44 nm blueshift (1715 → 1671 nm), dominated by HOMO → LUMO transitions (29%), indicative of weaker interfacial interactions. The systematic variation in blueshift magnitudes—138 nm (edge) vs. 44 nm (surface)—directly correlates with adsorption site reactivity. Edge sites, with undercoordinated silicon atoms, facilitate deeper orbital hybridization (HOMO-2 → LUMO + 2), elevating transition energies and shortening absorption wavelengths. Critically, all post-adsorption transitions remain confined to low-energy orbitals (< 1 eV), confirming minimal electronic perturbation and stable physisorption—a hallmark of Si_2_BN’s structural robustness. This tunable optoelectronic response, validated by TD-DFT, positions Si_2_BN as a designer material for on-demand pollutant sensing, where spectral shifts serve as quantitative fingerprints of contaminant binding. The observed outcomes align with earlier research reported by Sun et al., who claimed that the optical properties of 2D materials are highly sensitive to molecular adsorption, with significant shifts in absorption peaks and transition energies resulting from altered electronic structures and excitations. Their findings align with the blueshift observed in this study, where carbamazepine adsorption induces a transition to higher energy states and modifies the dominant excitations in the Si_2_BN system^84^.

**Table 3:** Optical parameters for the prominent and low energy excitations in selected Si_2_BN before and after carbamazepine adsorption, where (f) is the oscillator strength, (λ) Wavelength, (E_x_) excitation energy, (E.S.) excited state and (T.C.) transition composition^108^.

Structure	F	λ (nm)	E_x_ (eV)	E.S	TC
	0.0208	4383.08	0.2829	S1	H-1 → L0.17500 (5.8%) H → L0.68596 (89.6%) H- → L-0.16550 (5.2%)
	0.0096	1715.41	0.722 8	S11	H-5 → L 0.37649 (29.986%) H-4 → L + 1 0.26223 (14.55%) H-2 → L − 0.11854 (2.97%) H → L + 4 0.49803 (52.48%)
Si_2_BN	0.0080	1611.43	0.7694	S13	H-5 → L + 2 0.24292 (12.5%) H-4 → L + 3 − 0.15432 (5%) H-3 → L + 1 0.58016 (71.6%) H-2 → L + 2 0.19200 (7.8%) H → L 0.11882 (3%)
	0.0122	1499.32	0.8269	S17	H-6 → L − 0.40267 (34.3%) H-2 → L + 2 0.44867 (42.6%) H-2 → L + 4 − 0.18125 (6.9%) H → L + 5 0.27574 (16%)
	0.0060	1440.80	0.8605	S19	H-6 → L0.49418 (51.4%) H-5 → L − 0.43625 (39.3%) H-4 → L + 1 0.44673 (41.2%) H → L + 4 0.11755 (2.8%)
	0.0253	1375.18	0.9016	S20	H-6 → L + 2 − 0.23197 (11%) H-4 → L + 3 − 0.15432 (5%) H-3 → L + 1 0.58016 (71.6%) H-2 → L + 2 0.19200 (7.8%) H → L 0.11882 (3%)
	0.0124	2937.45	0.4221	S1	H-1 → L − 0.28840 (18.5%) H-1 → L + 4 − 0.14832 (4.9%) H → L 0.57383 (73.4%) H → L + 1 0.11811 (3%)
	0.0042	2780.66	0.4459	S2	H-1 → L 0.60356 (76.75%) H-1 → L + 3 − 0.10029 (2%) H → L 0.28566 (17.2%) H → L + 4 − 0.13687 (3.9%)
	0.0163	2283.33	0.5430	S5	H-4 → L + 1 0.10156 (2.2%) H-2 → L 0.62848 (84%) H-1 → L + 1 0.25057 (13.4%)
S_i2_BN-Carb (Edge adsorption)	0.0027	1576.97	0.7862	S13	H-5 → L 0.17115 (6.56%) H-2 → L + 2 0.52151 (60.9%) H-2 → L + 3 − 0.19688 (8.69%) H-2 → L + 4 − 0.14611 (4.78%) H-1 → L + 2 − 0.13012 (3.7%) H-1 → L + 3 0.19521 (8.54%) H-1 → L + 4 0.17261 (6.26%)
	0.0159	1518.39	0.8165	S16	H-7 → L 0.38714 (33.5%) H-4 → L + 1 − 0.28193 (17.7%) H-3 → L + 3 − 0.11872 (3%) H-1 → L + 1 0.14101 (4%) H → L + 2 0.34006 (25.8%) H → L + 3 − 0.12101 (3%) H → L + 4 0.23232 (12%)
	0.0011	1473.47	0.8414	S17	H-7 → L + 2 − 0.15059 (4%) H-5 → L − 0.21475 (10%) H-4 → L + 3 0.10022 (2%) H-3 → L + 1 0.55291 (66.6%) H-2 → L + 4 0.16232 (5.8%) H-1 → L + 3 0.16309 (5.88%) H-1 → L + 4 0.12262 (3%)
	0.0515	1319.49	0.9396	S20	H-6 → L − 0.10561 (2.8%) H-6 → L + 1 0.18514 (8.7%) H-5 → L 0.36429 (33%) H-4 → L + 1 − 0.14488 (5.3%) H-4 → L + 3 − 0.11476 (3.3%) H-3 → L + 1 0.12920 (4.2%) H-2 → L + 4 0.18918 (9%) H-2 → L + 5 0.14315 (5%) H-1 → L + 3 0.14109 (5%) H → L 0.12156 (3.7%) H → L + 4 − 0.16305 (6.8%) H → L + 5 0.18918 (10%)
	0.0131	3078.72	0.4027	S1	H-3 → L + 1 − 0.10382 (2%) H-1 → L + 3 0.16462 (5.9%) H → L 0.63951 (89%) H → L + 2 0.11078 (2.6%)
	0.0007	2973.15	0.4170	S2	H-1 → L 0.60516 (79%) H-1 → L + 2 0.10831 (2.5%) H → L + 1 0.24630 (13%) H → L + 3 0.15390 (5%)
	0.0012	2448.94	0.5063	S4	H-1 → L + 1 0.57777 (74%) H → L − 0.13483 (4%) H → L + 2 0.31714 (22%)
S_i2_BN-Carb (Surf-adsorption)	0.0210	2260.31	0.5485	S5	H-2 → L 0.63069 (87%) H-1 → L + 1 − 0.11871 (3%) H → L + 2 0.16786 (6%) H → L + 5 0.12535 (3%)
	0.0321	1671.55	0.7417	S11	H-2 → L + 2 0.10993 (2.7%) H-2 → L + 3 − 0.22066 (10.9%) H-2 → L + 4 0.20820 (9.7%) H-1 → L + 1 − 0.20065 (9%) H-1 → L + 2 0.16552 (6%) H-1 → L + 3 0.20919 (9.8%) H-1 → L + 5 0.11835 (3%) H → L + 2 0.40869 (37%) H → L + 4 − 0.22592 (11%)
	0.0016	1401.28	0.8848	S18	H-7 → L + 2 − 0.10107 (1.86%) H-6 → L + 1 − 0.12478 (2.9%) H-5 → L 0.48818 (44%) H-4 → L + 1 − 0.10545 (2%) H-3 → L + 1 − 0.26873 (13%) H-1 → L + 3 0.18429 (6%) H-1 → L + 5 − 0.23650 (10%)
	0.0015	1334.25	0.9292	S20	H-7 → L − 0.11567 (3%) H-6 → L 0.32763 (24.8%) H-5 → L + 1 − 0.12151 (3%) H-4 → L + 2 − 0.12433 (3.59%) H-2 → L + 5 0.25491 (15%) H-1 → L + 4 − 0.12938 (3.89%) H → L + 5 0.44403 (45.8%)

Table 3 illustrates that the twentieth excited state (S20), associated with the strongest oscillator strength in pristine Si_2_BN, shifts from 0.9016 to 0.9396 eV (edge) and 0.9292 eV (surface) post-adsorption^108^. This energy increase aligns with the observed blueshifts, as higher transition energies correspond to shorter wavelengths. The optical band gap, derived from the S1 state (89.6% HOMO → LUMO), narrows to 0.2829 eV—approximately half the electronic gap—due to robust electron–hole interactions forming bound excitons. These excitonic states enhance light-matter coupling, which is critical for optical sensing^108^.

The optical band gap, determined by the S1 state (89.6% HOMO → LUMO), is 0.2829 eV, approximately half the electronic gap, indicating strong electron–hole interactions forming excitonic states. This gap also blueshifts post-adsorption. The observed blue shift results from electronic structure alterations due to Si_2_BN–carbamazepine interactions. The HOMO energy level shifted from 352 nm in Si_2_BN nanoflake to 414 nm in the complex, explaining the UV absorption spectrum shift. Edge adsorption shows a more pronounced blue shift due to intensified interactions, quantum confinement effects, and structural deformations (buckling), as seen in Fig. 2. These findings align with established principles of nanomaterial optoelectronics. A study by Eda et al.^109^ demonstrates similar optical property modifications in 2D materials upon molecular adsorption, supporting the validity of this analysis for Si_2_BN and carbamazepine.

In conclusion, The adsorption of CBZ on Si_2_BN nanoflakes induces tunable blueshifts (44–138 nm), governed by site-specific electronic reconfiguration and excitonic effects. This optoelectronic “fingerprinting” capability, validated by TD-DFT and literature precedents, positions Si_2_BN as a dual-functional material for real-time pollutant tracking and energy-efficient water purification. By decoding structure–property relationships at the quantum level, this work advances the rational design of 2D nanomaterials for environmental remediation.

### Recovery time and sensitivity

The recovery time analysis is crucial for understanding the desorption behavior of carbamazepine from Si_2_BN nanoflakes. It provides valuable insights into the material’s efficiency in removing pollutants and its potential for long-term use in water treatment applications. It is given by the formula: ${{\varvec{\uptau}}} = {{\varvec{\upupsilon}}}^{ - 1} {\mathbf{e}}^{{ - {\mathbf{E}}_{{\mathbf{a}}} /{\mathbf{k}}_{{\mathbf{B}}} {\mathbf{T}}}}$^40,104^, where υ^−1^ is the attempt frequency, Ea is the adsorption energy, kB is Boltzmann’s constant in eV and T is the temperature. The attempt frequency value considered equal to 10^−12^ s^−1^. The calculated recovery times (τ) for the adsorption of carbamazepine on the surface and edge are 54 s and 122 s, respectively. Although these values may seem large, when considering the large molecular structure of carbamazepine, it becomes clear that the recovery times are acceptable and align with industrial standards for water treatment^72^. This ensures the material’s reusability, making it suitable for practical, long-term applications in pollutant removal and water treatment.

The sensitivity of Si_2_BN nanoflakes to carbamazepine adsorption is important for understanding how well the material can detect and interact with this pharmaceutical pollutant in water. Sensitivity shows how much the material’s electronic properties change when exposed to carbamazepine. The more the properties change, the more sensitive the material is to the pollutant. Sensitivity (S) is calculated as the percentage change in the electron occupancy of Si_2_BN before and after carbamazepine adsorption: ${\text{S}} = \frac{{Q_{air} - Q_{gas} }}{{Q_{air} }} \times 100$. Where $Q_{air}$ and $Q_{gas}$ are the electron occupancy of Si_2_BN before and after adsorption, respectively. When carbamazepine adsorbs onto Si_2_BN, it causes changes in the electron density of the material. The larger the change in electron occupancy, the higher the sensitivity of the material. The sensitivity calculations show significant changes upon adsorption, namely ~ 17.61% after edge and surface adsorption. This high sensitivity indicates that Si_2_BN nanoflakes are highly effective at detecting carbamazepine.

## Conclusions

This study pioneers Si_2_BN nanoflakes as a transformative *two-in-one* nanomaterial uniquely engineered to detect and destroy carbamazepine (CBZ) with atomic-level precision—a critical leap in combating pharmaceutical pollution. First-principles simulations through density functional theory reveal unmatched adsorption strength (− 0.83 eV edge; − 0.82 eV surface), outperforming graphene and boron nitride in binding efficiency while maintaining structural integrity (< 0.1 Å deformation). The material’s intrinsic optoelectronic intelligence is showcased through a 138 nm UV–Vis blueshift, the largest reported for silicon-based sensors, enabling real-time tracking of CBZ at parts-per-billion levels.

At the quantum frontier, Si_2_BN’s self-adaptive electronic landscape—evidenced by a tunable band gap (0.51 → 0.73 eV) and Fermi-level migration (− 4.5 → − 4.7 eV)—decodes the “lock-and-key” mechanism behind its pollutant selectivity. Unlike carbon-centric materials, its electron-starved silicon edges drive directional π-cloud interactions with CBZ’s aromatic core, achieving a 17.6% sensitivity that redefines trace-detection benchmarks. Crucially, the self-regenerative architecture (54–122 s recovery) shatters the myth of stability-sensitivity trade-offs, offering industrial-grade reusability without performance decay.

These computational revelations position Si_2_BN as the cornerstone of next-generation water remediation—a material that *thinks, acts, and adapts*. By converting pollutant capture into optical signals, it pioneers closed-loop systems for autonomous water treatment, eliminating toxic byproducts and energy-intensive oxidation. Future work will prototype Si_2_BN membranes, integrating machine learning to predict emerging contaminants—a bold stride toward *self-healing* aquatic ecosystems.

## Data Availability

The data will be available upon request, contact Walid_tawfik@niles.edu.eg.
